# Clinical characteristics and the prognosis of diabetic foot in Tibet: A single center, retrospective study

**DOI:** 10.1515/biol-2022-0740

**Published:** 2023-10-17

**Authors:** Xiaoyong Yuan, Difei Lu, Shuyou Meng, Lihui Yang, Geheng Yuan, Xin Qi, Bing Wen, Junqing Zhang, Xiaohui Guo

**Affiliations:** Department of Endocrinology, Peking University First Hospital, No. 8 Xishiku Street, Xicheng District, Beijing 100034, China; Department of Endocrinology, Tibet Autonomous Region People’s Hospital, Lhasa, Tibet Autonomous Region, 850010, China; Department of Plastic and Reconstructive Surgery, Peking University First Hospital, Beijing 100034, China

**Keywords:** region differences, diabetic foot, infection, amputation, death rate

## Abstract

The objective of this study was to explore the clinical characteristics and prognosis of diabetic foot in hospitalized patients with diabetes in Tibet. To achieve that, patients hospitalized in People’s Hospital of Tibet Autonomous Region and diagnosed with diabetic foot ulcer (DFU) from January 1, 2016 to December 31, 2020 were enrolled in the study, and DFU cases of Peking University First Hospital were collected as control group. Analysis and comparison of clinical characteristics of DFU in plateau and plain areas were conducted. Normal distribution data or non-normal distribution data between groups were analyzed by *t*-test analysis or the nonparametric Mann–Whitney *U* test, and categorical variants were compared by Chi-square of Pearson. A total of 54 DFU cases were enrolled in the study in the People’s Hospital of Tibet Autonomous Region (Tibet group for short). Males accounted for 83.3% (45 cases) in Tibet group, which was higher than that of Peking University First Hospital (Beijing group for short), which accounted for 67.0%. Compared with the DFU patients in the Beijing group, the Tibet group was younger (58.11 ± 12.25 years vs 64.18 ± 11.37 years, *P* < 0.05), with a shorter disease duration (7.00 years vs 12.00 years, *P* < 0.05). In contrast, alcohol consumption was higher in the Tibet group (44.4 vs 27.4%, *P* < 0.05), and the number of patients with smoking habit was higher in the Beijing group (29.6 vs 43.7%, *P* < 0.05). The Tibet group had higher HbA1c (10.2 vs 8.7%, *P* < 0.05) and lower DFU proportion (22.2 vs 44.2%, *P* < 0.05). There was no statistically significant difference in the proportion of moderate to severe infections between the two groups (58.5 vs 59.6%, *P* = 0.887). Leukocytes (6.75 × 10^9^/L vs 8.72 × 10^9^/L, *P* < 0.05) and neutrophils (4.07 × 10^9^/L vs 6.26 × 10^9^/L, *P* < 0.05) in Tibet group were lower. Although the DFU amputation rate in the Tibet group was lower than that in the Beijing group (9.3 vs 29.8%, *P* < 0.05), there was no statistically significant difference between the two groups in terms of treatment cost, hospital stay, and mortality. In conclusion, patients with DFU in Tibet had a smaller age, shorter duration of diabetes, and more male predominance. The proportions of gangrene and amputation were lower in Tibet, with gangrene accounting for 80% of all amputees.

## 1 Introduction

Diabetic foot ulcer (DFU) is the leading cause of non-traumatic amputation, which is also one of the most common complications leading to death in patients with diabetes mellitus (DM). According to a community-based study in northwestern United Kingdom [1], the annual incidence of foot ulcer was approximately 2.2%. The lifetime risk of DFU in diabetic patients was estimated to be 19–34%, and the recurrence rate was estimated to be approximately 40% within 1 year of ulcer healing and up to 65% within 5 years [2]. Despite striking data, the clinical implications for the foot ulcers remain limited. Approximately 30.5% of patients with different types of diabetic foot infection underwent different degrees of amputation [3]. A population-based cohort study in the United Kingdom demonstrated a 42% mortality rate associated with DFU. Within 5 years, the risk of death among DFU patients was 2.5 times that of diabetic patients without DFU [4]. Meanwhile, expenditure on direct healthcare for DFU was estimated to be $176 billion in 2012, about one-third of the total cost of diabetic care [5]. Based on the recent data from the UK, it was conservatively estimated that the annual cost of DFU in 2019 was £900 million, which reached approximately 1% of the total budget of the National Health Service [6].

Therefore, effective strategies for foot complications in diabetes patients are urgently needed to improve the poor clinical outcomes. However, few previous studies on DFU rarely involved plateau regions. The unique climate of plateau areas, including low temperature, hypoxia, and dryness, may have certain effects on wound healing, amputation rate, and mortality. According to Wanle Qi’s report, wound healing of elderly DFU patients in plateau area were delayed, accounting for 51.9% of the total chronic refractory wounds [7]. There was a lack of research on the clinical features of DFU in the plateau area. Therefore, this study aims to compare the differences in clinical characteristics and prognosis of DFU in Tibet and Beijing, and then further investigate possible confounders.

## 2 Materials and methods

### 2.1 Settings and participants

DM was diagnosed when fasting blood glycemia ≥126 mg/dL or glycosylated hemoglobin (A1c) ≥6.5% according to standards of medical care in diabetes from American Diabetes Association [8]. Patients with previous diagnosis of DM and registered in medical records were also considered as the diagnosis of DM. DFU was considered when the patients were previously diagnosed as diabetic foot infection or ischemic DFU with medical records. Wagner classification [9] was used to evaluate the severity of diabetic foot. Moderate infection and severe infection for DFU was defined according to IDSA criteria [10]. The exclusion criteria were as follows: (1) basic information was incomplete, (2) admitted to hospital for a same ulcer (In other words, if a patient had a hospitalization for a DFU and was subsequently readmitted for the exact same DFU within the specified timeframe, they would be excluded from our analysis.), and (3) ulcer had healed when admission.

Patients with DFU who met the above criteria and were hospitalized in the duration from January 1, 2016 to December 31, 2020 in Tibet Autonomous Region People’s Hospital were enrolled in the study as Tibet group. Patients with DF and hospitalized in Peking University First Hospital between January 1, 2010 and December 31, 2014 were enrolled as control group. We identified all DF subjects through medical records, and data collection was performed using the paper version of case report form.

The study protocol was approved by Ethics Committees of Tibet Autonomous Region People’s Hospital and was in accordance with Helsinki Declaration principles. Our study was approved by the Ethics Committees of Tibet Autonomous Region People’s Hospital (No. ME-TBHP-21-030).


**Informed consent:** Informed consent has been obtained from all individuals included in this study.
**Ethical approval:** The research related to human use has been complied with all the relevant national regulations, institutional policies and in accordance with the tenets of the Helsinki Declaration, and has been approved by the Ethics Committees of Tibet Autonomous Region People s Hospital (No. ME-TBHP-21-030).

### 2.2 Variables

The clinical characteristics of DF patients were collected as follows: the type of diabetes, DM duration, type of anti-diabetic drugs, complications of DM, history of smoking and drinking, body mass index (BMI), body temperature, gangrene, and Wagner classification when admission. Data of clinical outcomes were accumulated including treatment, the rate of amputation, rate of death, length of hospital stay, and cost. Laboratory results including white blood cell count (WBC), neutrophils (Net), red blood cell count (RBC), hemoglobin (Hb), C-reactive protein (CRP), procalcitonin (PCT), serum albumin (Alb), uric acid (UA), fasting blood glucose (Glu), glycosylated hemoglobin (HbA_1c_), triglyceride (TG), total cholesterol (TCHO), high density lipoprotein cholesterol (HDL-c), and low density lipoprotein cholesterol (LDL-c) were also collected.

### 2.3 Statistical analysis

Variables in line with normal distribution were described as mean ± standard deviation, and were compared using Student’s *t*-test between groups. Categorical variables were presented as percentage, and were compared using Chi-square of Pearson between groups. Data were analyzed using Stata version 25.0. *P* < 0.05 was considered as statistical significance.

## 3 Results

In this study, there were 54 inpatients with DFU in Tibet Autonomous Region People’s Hospital, and 215 cases in Peking University First Hospital. In the Tibet group, type 2 diabetes patients accounted for 98.1% (53/54), men accounted for 83.3% (45/54), the mean age was 58.11 ± 12.25 years, and the median duration of DM was 7 years. In the Beijing group, males accounted for 67.0% (144/215), the mean age was 64.18 ± 11.37 years, and the median duration of DM was 12 years (Table 1).

**Table 1:** Characteristics comparison of DFU patients between Tibet Autonomous People’s Hospital (Tibet) and Peking University First Hospital (Beijing)

	Tibet (*X* ± *D*, *M*(*Q*1,*Q*3), *n*(%))	Beijing (*X* ± *D*, *M*(*Q*1,*Q*3), *n*(%))	*t/Z/χ* ^*2*^	*P*
Number of patients	54	215		
General characteristics				
Age (years)	58.11 ± 12.25	64.18 ± 11.37	−3.454	0.001
Course of DM (years)	7.00(4.00, 11.50)	12.00(8.00, 20.00)	−4.039	<0.001
History of smoking	16(29.6%)	94(43.7%)	11.690	0.003
History of drinking	24(44.4%)	59(27.4%)	10.749	0.005
BMI (kg/m^2^)	24.43 ± 3.55	24.67 ± 3.66	−0.338	0.736
Characteristics of disease				
Increased body temperature (>37.3℃)	1(1.9%)	19(8.8%)	3.060	0.080
Thanatosis	12(22.2%)	95(44.2%)	9.764	0.002
Wagner classify				
Level 1	6(11.1%)	32(14.9%)	0.506	0.477
Level 2	16(29.6%)	44(20.5%)	2.092	0.148
Level 3	20(37.0%)	32(14.9%)	13.583	0.000
Level 4	12(22.2%)	75(34.9%)	3.162	0.075
Level 5	0(0.0%)	8(3.7%)	2.071	0.150
Infection situation				
Moderate infection	24(45.3%)	95(50.5%)	0.456	0.500
Severe infection	7(13.2%)	17(8.1%)	1.038	0.253
Moderate to severe infection	31(58.5%)	112(59.6%)	0.020	0.887
Laboratory characteristics				
WBC (10^9^/L)	6.75(5.21, 9.98)	8.72(6.70, 11.37)	−2.937	0.003
Net (10^9^/L)	4.07(2.93, 7.25)	6.26(4.47, 8.75)	−3.272	0.001
WBC > 10 × 10^9^/L	12(23.1%)	75(35.7%)	3.001	0.083
Net > 70%	20(38.5%)	132(62.9%)	10.184	0.001
CRP (mg/L)	14.53(3.21, 90.03)	47.05(9.49, 104.87)	−1.431	0.152
PCT (ng/ml)	0.19(0.05, 0.51)	0.12(0.06, 0.32)	−0.361	0.718
RBC (10^12^/L)	4.57 ± 1.05	3.83 ± 0.73	4.785	<0.001
Hb (g/L)	138.31 ± 34.79	113.69 ± 23.06	4.845	<0.001
Alb (g/L)	31.67 ± 6.76	33.13 ± 5.63	−1.617	0.107
UA (μmol/L)	337(278, 438)	292(234, 374)	−3.352	0.001
Glu (mmol/L)	8.55(6.30, 12.03)	7.23(5.54, 10.66)	−1.993	0.046
HbA1c (%)	10.2(7.8, 13.0)	8.7(7.3, 10.6)	−3.027	0.002
TG (mmol/L)	1.03(0.79, 1.29)	1.16(0.84, 1.56)	−1.701	0.089
TCHO (mmol/L)	3.63(3.12, 4.55)	3.77(3.22, 4.48)	−0.589	0.556
HDL (mmol/L)	0.96 ± 0.28	0.85 ± 0.25	2.648	0.009
LDL (mmol/L)	2.23(1.65, 2.95)	2.34(1.91, 2.97)	−0.959	0.338
Outcome features				
The amputation rate	5(9.3%)	64(29.8%)	9.518	0.002
Death rate	1(1.9%)	4(1.9%)	0.000	0.997
Hospitalization expenses (RMB)	20149.61(14834.36, 32739.27)	19029.85(10348.14, 44248.05)	−0.990	0.322
Length of hospital stay (days)	13(11, 17)	16(12, 25)	−2.446	0.014

In contrast, the incidence of DFU combined with coronary heart disease in Tibet was lower than that in Beijing (0 vs 39.7%, *P* < 0.001). The incidence of other complications in Tibet was also lower than that in Beijing, including peripheral arterial disease (9.3 vs 86.2%, *P* < 0.001), peripheral neuropathy (13.0 vs 64.8%, *P* < 0.001), cerebrovascular disease (5.6 vs 64.0%, *P* < 0.001), and diabetic retinopathy (24.1 vs 53.3%, *P* < 0.001). There was no statistically significant difference in the complication rate of diabetic nephropathy between DFU patients in Tibet and Beijing (42.6 vs 50.3%, *P* = 0.329), as well as those with former diabetic foot (18.5 vs 31.8%, *P* = 0.055). Fewer patients were diagnosed with peripheral neuropathy in Tibet than in Beijing (11.1 vs 47.4%, *P* < 0.001). The incidence of peripheral arterial disease was similar in the two groups (92.6 vs 84.2%, *P* = 0.113).

There were fewer patients with gangrene (22.2 vs 44.2%, *P* = 002) or amputation (9.3 vs 29.8%, *P* = 0.002) in Tibet than in Beijing. For DFU patients in Tibet and Beijing, there was no statistically significant difference in moderate–severe infection (58.5 vs 59.6%, *P* = 0.887). Both the WBC elevation rate (38.5 vs 62.9%, *P* = 0.0001) and Net elevation rate (38.5 vs 62.9%, *P* = 0.0001) in Tibet were lower than those in Beijing. The death rates of DFU in the two areas were of no significance (1.9 vs 1.9%, *P* = 0.997, Tables 2 and 3).

**Table 2:** Characteristics of amputation in DFU patients in the People’s Hospital of Tibet Autonomous Region

	Non-amputation patients (*X* ± *D*, *M*(*Q*, *R*), *n*(%))	Amputation patients (*X* ± *D*, *M*(*Q*, *R*), *n*(%))	*t/Z/χ* ^*2*^	*P*
Age (age)	59.16 ± 12.13	47.80 ± 8.73	2.033	0.047
Course of DM (years)	7.50(4.25, 12.00)	5.00(0.50, 8.00)	−1.511	0.131
History of smoking	14(28.6%)	2(40.0%)	0.284	0.594
History of drinking	22(44.9%)	2(40.0%)	0.044	0.834
Thanatosis	8(16.3%)	4(80.0%)	10.643	0.001
Wagner classify				
Level 1	6(12.2%)	0(0.0%)	0.689	0.407
Level 2	16(32.7%)	0(0.0%)	2.320	0.128
Level 3	19(38.8%)	1(20.0%)	0.686	0.408
Level 4	8(16.3%)	4(80.0%)	10.643	0.001
Level 5	0(0.0%)	0(0.0%)		
Infection situation				
Moderate infection	21(57.1%)	3(60.0%)	1.542	0.214
Severe infection	6(12.2%)	1(20.0%)	0.525	0.469
Moderate to severe infection	27(55.1%)	4(80.0%)	3.070	0.080
Laboratory characteristics				
WBC (10^9^/L)	6.75(5.21, 9.98)	7.00(5.38, 19.70)	−0.446	0.655
Net (10^9^/L)	4.07(2.84, 7.25)	4.56(3.23, 16.38)	−0.601	0.548
RBC (10^12^/L)	4.56 ± 1.07	4.68 ± 0.90	−0.216	0.830
Hb (g/L)	138.27 ± 35.50	138.75 ± 28.65	−0.026	0.979
Alb (g/L)	31.34 ± 6.94	34.86 ± 3.83	−1.111	0.272
UA (μmol/L)	334(272, 438)	413(325, 479)	−1.133	0.257
Glu (mmol/L)	9.04(6.45, 12.78)	6.30(5.76, 7.17)	−2.134	0.033
HbA1c (%)	10.40(7.58, 10.25)	10.1(9.15, 10.25)	−0.506	0.613
TG (mmol/L)	0.98(0.77, 1.25)	0.118(1.00, 1.43)	−1.106	0.269
TCHO (mmol/L)	3.57(3.07, 4.55)	3.71(3.43, 4.48)	−0.468	0.640
HDL (mmol/L)	0.980 ± 0.29	0.750 ± 0.13	1.592	0.119
LDL (mmol/L)	2.21(1.62, 2.95)	2.59(2.30, 2.95)	−1.144	0.253

**Table 3:** Characteristics comparison of amputations in DFU patients between the People’s Hospital of Tibet Autonomous Region (Tibet) and Peking University First Hospital (Beijing)

	Tibet (*X* ± *D*, *M*(*Q*, *R*), *n*(%))	Beijing (*X* ± *D*, *M*(*Q*, *R*), *n*(%))	*t/Z/χ* ^*2*^	*P*
Age (age)	47.80 ± 8.73	64.70 ± 9.73	−3.763	<0.001
Course of DM (years)	5.00(0.50, 8.00)	16.50(9.50, 20.00)	−2.834	0.005
History of smoking	2(40.0%)	21(32.8%)	0.385	0.825
History of drinking	2(40.0%)	13(20.3%)	1.310	0.520
Thanatosis	4(80.0%)	9(14.1%)	13.187	<0.001
Wagner classify				
Level 1	0(0.0%)	1(1.6%)	0.079	0.778
Level 2	0(0.0%)	4(6.3%)	0.332	0.565
Level 3	1(20.0%)	10(15.6%)	0.066	0.797
Level 4	4(80.0%)	39(60.9%)	0.718	0.397
Level 5	0(0.0%)	6(9.4%)	0.513	0.474
Infection situation				
Moderate infection	3(60.0%)	48(75.0%)	0.098	0.754
Severe infection	1(20.0%)	6(9.4%)	0.963	0.326
Moderate to severe infection	4(80.0%)	54(84.4%)	0.368	0.544
Laboratory characteristics				
WBC (10^9^/L)	7.00(5.38, 19.70)	9.20(7.12, 11.60)	−0.887	0.375
Net (10^9^/L)	4.56(3.23, 16.38)	7.11(5.31, 9.24)	−1.085	0.278
RBC (10^12^/L)	4.68 ± 0.90	3.67 ± 0.70	2.745	0.008
Hb (g/L)	138.75 ± 28.65	107.03 ± 22.30	2.718	0.008
Alb (g/L)	34.86 ± 3.83	31.76 ± 5.06	1.334	0.187
UA (μmol/L)	413(325, 479)	257(203, 363)	−2.411	0.016
Glu (mmol/L)	6.30(5.76, 7.17)	7.84(5.79, 11.78)	−1.059	0.290
HbA1c (%)	10.1(9.15, 10.25)	8.8(7.6, 10.4)	−0.920	0.358
TG (mmol/L)	1.18(1.00, 1.43)	1.11(0.79, 1.41)	−0.516	0.606
TCHO (mmol/L)	3.71(3.43, 4.48)	3.54(2.97, 4.19)	−0.670	0.503
HDL (mmol/L)	0.750 ± 0.13	0.760 ± 0.22	−0.082	0.935
LDL (mmol/L)	2.59(2.30, 2.95)	2.34(1.89,3.02)	−0.888	0.374

## 4 Discussion

Tibet is a special area characterized by several local diseases or special features of common diseases due to hypoxia and severe cold in high altitude areas, for instance less infection from resistant bacteria [11], higher prevalence of parasites [12], higher prevalence of hypertension [13], and endemic high altitude polycythemia [14]. In our study, the clinical features of diabetic foot in Tibet were different from that in plain areas such as Beijing. Compared with diabetic patients in Beijing, patients in Tibet were more likely to develop DFU in a relatively smaller age (58.11 years vs 64.18 years, *P* = 0.001), and the youngest DFU patient in Tibet was only 34 years old. Meanwhile, patients in Tibet presented with 5 years earlier in diabetes duration at the onset of DFU compared with patients in Beijing (7.00 years vs 12.00 years, *P* < 0.001).

Diabetic patients often suffer from the stenosis of lower extremity artery due to long-term hyperglycemia, which would lead to extremity ischemia. Harsh climate in plateau combining severe cold and hypoxia could aggravate lower extremity ischemia [15] furthermore leading to lower extremity ulcers or gangrene. The severe cold climate could lead to diminished sensitivity of peripheral sensory nerve in diabetic patients [16], which could further bring about frostbite or scald. Therefore, DFU patients in Tibet are younger and with shorter duration of diabetes. In addition, poor glycemic control may also contribute to the earlier onset of DFU in Tibet, which was demonstrated by the phenomenon that the HbA1c of DFU patients in Tibet increased by 1.5% (10.2 vs 8.7%, *P* = 0.002) compared with patients in Beijing.

In terms of the severity of diabetic foot, DFU in Tibet was mild presented compared with Beijing. The proportion of moderate-to-severe infection of DFU in these two areas was similar (80.0 vs 84.4%, *P* = 0.544), while the inflammatory indicators reflecting the severity of infection including WBC, Net, and rate of elevated WBC and elevated Net, in Tibet, were lower than Beijing. It was speculated that the milder infection might be related to the special environment in Tibet. Although people in Tibet live in poor sanitary conditions with limited frequency of self-examination, and hypoxia and severe cold abated the immune response of them, which could increase the possibility of skin and soft tissue infection. However, the harsh environment in plateau area also restricted the survival and growth of pathogens [17], especially for those infecting skin and soft tissues. Therefore, the infection rate of DFU in Tibet was similar to that in Beijing, but the inflammatory indicators were lower. On the other hand, DFU in Tibet showed a reduced risk of gangrene (22.2 vs 44.2%, *P* < 0.001). Gangrene is caused by vascular occlusion of lower extremities, while altered glucose and lipid metabolism are risk factors for vascular disease. The HbA1c and lipid profile were revealed to be in poor control in DFU patients in Tibet, which was inconsistent with the situation of the small proportion of gangrene. Taking the population characteristics of DFU in Tibet into account, the smaller age, shorter course of disease, and low smoking rate could be main contributors to the low risk of gangrene in Tibet.

With regard to the clinical outcomes, the mortality rate of DFU in the two areas was insignificant (1.9 vs 1.9%, *P* = 0.997), while the amputation rate in Tibet was significantly lower than that in Beijing (9.3 vs 29.8%, *P* = 0.002). In terms of mortality, it has commonly been assumed that the main cause of death in DFU was cardiovascular and cerebrovascular diseases or serious infections [9]. The harsh environment of the plateau might not be a significant influencing factor of cardiovascular lesions for DM patients, thus the mortality rate was comparable of the two areas. A possible explanation for the reduced amputation rate in Tibet might be the smaller age of DFU patients in Tibet the shorter duration of DM. Also, gangrene accounted for 80% of DFU with amputation in Tibet, which was higher than Beijing. Therefore, the lower rate of amputation in Tibet could be the result of fewer gangrene and milder infection. Thus, the younger age, the shorter duration of DM, lower smoking rate, the lower proportion of gangrene, and the milder infection may contribute to the decrease of the overall amputation rate in Tibet.

There were several limitations in this study. First, this was a retrospective study, and was insufficient for causal inferences. Therefore, we could only provide the possible influencing factors for characteristics of DFU in Tibet. Second, the number of DFU patients in Tibet was small, which may lead to bias in subgroup analysis.

Furthermore, for practical considerations, we chose Beijing as the control group. The First Hospital of Beijing is a well-established institution with a larger patient population, making it easier to collect an adequate number of DFU cases for comparison. Additionally, the hospital possesses specialized knowledge in treating DFUs, ensuring standardized care protocols. We understand the concerns regarding the differences between Beijing and Tibet potentially introducing confounders and limiting the generalizability of our findings. The variations in demographics, culture, environment, and economic development between these two regions are indeed important factors to consider. However, it is worth noting that there is a scarcity of studies investigating diabetic foot in Tibet. Our study fills this gap in the literature by providing valuable insights into the clinical characteristics and prognosis of diabetic foot in this specific region. Despite the limitations associated with the choice of the comparison group, we believe that our research still contributes to the understanding of diabetic foot in Tibet.

## 5 Conclusions

Our study compared the clinical characteristics and outcomes of DFU patients in Tibet and Beijing. The results showed that DFU patients in Tibet were younger, had a shorter duration of diabetes, and had a lower incidence of complications such as coronary heart disease, peripheral arterial disease, peripheral neuropathy, cerebrovascular disease, and diabetic retinopathy compared to those in Beijing. Additionally, DFU patients in Tibet had a lower rate of gangrene and amputation, milder infection, and comparable mortality rates. These differences could be attributed to the unique environment and population characteristics of Tibet. However, the study has limitations, including its retrospective nature and a small sample size in Tibet, highlighting the need for further research to validate these findings.

## References

[1] Abbott CA, Carrington AL, Ashe H, Bath S, Every LC, Griffiths J, et al. The North-West Diabetes Foot Care Study: incidence of, and risk factors for, new diabetic foot ulceration in a community-based patient cohort. Diabet Med. 2002 May;19(5):377–84. 10.1046/j.1464-5491.2002.00698.x. PMID: 12027925. 12027925

[2] Armstrong DG, Boulton AJM, Bus SA. Diabetic foot ulcers and their recurrence. N Engl J Med. 2017 Jun 15;376(24):2367–75. 10.1056/NEJMra1615439. PMID: 28614678. 28614678

[3] Sen P, Demirdal T, Emir B. Meta-analysis of risk factors for amputation in diabetic foot infections. Diabetes Metab Res Rev. 2019 Oct;35(7):e3165. 10.1002/dmrr.3165. Epub 2019 May 6. PMID: 30953392. 30953392

[4] Walsh JW, Hoffstad OJ, Sullivan MO, Margolis DJ. Association of diabetic foot ulcer and death in a population-based cohort from the United Kingdom. Diabet Med. 2016 Nov;33(11):1493–8. 10.1111/dme.13054. Epub 2016 Jan 10. PMID: 26666583. 26666583

[5] Skrepnek GH, Mills JL Sr, Lavery LA, Armstrong DG. Health care service and outcomes among an estimated 6.7 million ambulatory care diabetic foot cases in the U.S. Diabetes Care. 2017 Jul;40(7):936–42. 10.2337/dc16-2189. Epub 2017 May 11. PMID: 28495903. 28495903

[6] Kerr M, Barron E, Chadwick P, Evans T, Kong WM, Rayman G, et al. The cost of diabetic foot ulcers and amputations to the National Health Service in England. Diabet Med. 2019 Aug;36(8):995–1002. 10.1111/dme.13973. Epub 2019 Jun 5. PMID: 31004370. 31004370

[7] Ma Z, Mejia Z, Qi W, Li J, Gong Y. Epidemiological investigation and analysis of elderly patients with chronic refractory wounds in plateau area. Clin J Injury Repair Wound Healing(Electronic Ed). 2021 Feb;16(1):44–9.

[8] American Diabetes Association, 15. Diabetes care in the hospital: standards of medical care in diabetes 2021. Diabetes Care. 2021;44:S15–33. 10.2337/dc21-S002. 33298426

[9] Wagner FW Jr. The dysvascular foot: a system for diagnosis and treatment. Foot Ankle. 1981 Sep;2(2):64–122. 10.1177/107110078100200202. PMID: 7319435. 7319435

[10] Hinchliffe RJ, Forsythe RO, Apelqvist J, Boyko EJ, Fitridge R, Hong JP, et al. Guidelines on diagnosis, prognosis, and management of peripheral artery disease in patients with foot ulcers and diabetes (IWGDF 2019 update). Diabetes Metab Res Rev. 2020 Mar;36 Suppl 1:e3276. 10.1002/dmrr.3276. Epub 2020 Jan 20. PMID: 31958217. 31958217

[11] Meilang Q, Li R, Wu XM, Shang Y, Ning P, Bao J, et al. Clinical and etiological characteristics of community-acquired pneumonia at high altitudes in Tibet, China. Chin Med J (Engl). 2020 Nov;134(6):749–51. 10.1097/CM9.0000000000001166. PMID: 33725710; PMCID: PMC7989991. PMC7989991 33725710

[12] Bianba ZM. Survey of human intestinal protozoal infections in Tibet Autonomous Region, 2015. Zhongguo Xue Xi Chong Bing Fang Zhi Za Zhi. 2020 Jun;32(5):502–5. Chinese. 10.16250/j.32.1374.2020095. PMID: 33185062. 33185062

[13] Song C, Chongsuvivatwong V, Zhu Luo Bu O, Ji D, Sang Zhuo Ma B, Sriplung H. Relationship between hypertension and geographic altitude: a cross-sectional survey among residents in Tibet. J Int Med Res. 2020 Feb;48(2):300060520903645. 10.1177/0300060520903645. PMID: 32090671; PMCID: PMC7111057. PMC7111057 32090671

[14] Deng R, Labasangzhu, Zhaxideji, Wang G, Hong P, Li J, et al. Illness prevalence rate in Tibet, China: data from the 2018 national health service survey. BMC Public Health. 2020 Jun;20(1):955. 10.1186/s12889-020-08960-7. PMID: 32552694; PMCID: PMC7302388. PMC7302388 32552694

[15] Houdas Y, Deklunder G, Lecroart JL. Cold exposure and ischemic heart disease. Int J Sports Med. 1992 Oct;13(Suppl 1):S179–81. 10.1055/s-2007-1024632. PMID: 1483767. 1483767

[16] Herrera E, Sandoval MC, Camargo DM, Salvini TF. Motor and sensory nerve conduction are affected differently by ice pack, ice massage, and cold water immersion. Phys Ther. 2010 Apr;90(4):581–91. 10.2522/ptj.20090131. Epub 2010 Feb 25. PMID: 20185615. 20185615

[17] Liu K, Hou J, Liu Y, Hu A, Wang M, Wang F, et al. Biogeography of the free-living and particle-attached bacteria in Tibetan lakes. FEMS Microbiol Ecol. 2019 Jul;95(7):fiz088. 10.1093/femsec/fiz088. PMID: 31183497. 31183497

